# Detrimental effect of Hypoxia-inducible factor-1α-induced autophagy on multiterritory perforator flap survival in rats

**DOI:** 10.1038/s41598-017-12034-x

**Published:** 2017-09-18

**Authors:** Long Wang, Zhicheng Jin, Jieke Wang, Shao Chen, Li Dai, Dingsheng Lin, Lingfeng Wu, Weiyang Gao

**Affiliations:** 1 Department of Hand and Plastic Surgery, The Second Affiliated Hospital and Yuying Children’s Hospital of Wenzhou Medical University, Wenzhou, China; 2 Department of Orthopedics, The Fifth Affiliated Hospital and Central Hospital of Lishui City of Wenzhou Medical University, Lishui, China

## Abstract

Hypoxia-inducible factor-1α (HIF-1α) plays a key role in angiogenesis, improves flap survival, and activates autophagy. The effect of HIF-1α-induced autophagy is still debatable. Thus, we investigated the effect of HIF-1α-induced autophagy on multiterritory perforator flap survival. In this study, 99 male Sprague-Dawley rats received multiterritory perforator flap procedure and were divided into three groups with 33 each. The dimethyloxalylglycine (DMOG) plus 3-methyladenine (3-MA) group received intraperitoneal injection of DMOG (40 mg/kg) and 3-MA (10 mg/kg). The DMOG group and control group received comparative DMOG and saline respectively. On postoperative day (POD) 7, HIF-1α’s activities of flap survival and perfusion improvement were confirmed in DMOG group, however, its positive effects were further enhanced by co-administration of autophagy inhibitor, 3-MA. On POD 1, vascular endothelial growth factor, mean microvascular density and blood perfusion were not affected by HIF-1α up-regulation or autophagy inactivation. However, HIF-1α-induced autophagy augments apoptosis and oxidative stress. The increased level of apoptosis and oxidative stress was reversed by 3-MA and resulted in further flap survival improvement. In conclusion, HIF-1α-induced autophagy has a detrimental effect on multiterritory perforator flap survival and the flap survival was determined by the combined effects of ischemia and reperfusion injury.

## Introduction

A perforator flap involves a cutaneous perforator artery of 0.5 mm or greater without main vessel^1^. Both clinical and experimental studies demonstrated that necrosis always occurred at the dynamic territory boundary and at the potential territory^2–4^. With increase in size of the skin defect caused by severe trauma, burn and so on, a larger perforator flap including potential territory to cover the huge skin defect should be designed. Thus, it is necessary to find an effective method to increase the multiterritory perforator flap survival.

Hypoxia-inducible factor-1 (HIF-1) is a heterodimeric transcription factor consisting of oxygen sensitive α subunit and constitutively expressed β subunit. HIF-1α is induced by hypoxia, whereas HIF-1β is not. Under normoxic conditions, the synthetic HIF-1α protein is continuously and rapidly degraded via ubiquitin proteasome pathway after hydroxylation by HIF prolyl hydroxylases. However, hydroxylation of HIF-1α decreases during hypoxia or ischemia, leading to HIF-1α stabilization in the cytoplasm. After translocation into the nucleus, HIF-1α recruits HIF-1β and induces expression of target proteins^5–7^. Some authors showed that increased expression of HIF-1α could improve flap survival via vascularization^8,9^. And HIF-1α still demonstrated a positive effect on flap survival even in aged or diabetic mice^9,10^.

Recently, several experts reported that HIF-1α could regulate autophagy^11–13^. Autophagy is a self-catabolic process of damaged or dysfunctional cellular components which are recycled for providing energy and nutrients. Thus, the process of autophagy exerts a beneficial effect on cell survival function during nutrition deprivation or stress. However, excessive autophagic activity may play a detrimental role and induces cell death^14,15^. For example, activation of autophagy protects injured tissues during retinal detachment and spinal cord injury^12,16^. Even though the activated autophagy during the period of ischemia could improve the results of heart or brain ischemia/reperfusion injury, overexpression of the autophagy impairs the heart and brain during the period of reperfusion^17–19^. It still remains unclear whether autophagy is required for flap survival, but our study hypothesized that HIF-1α-induced autophagy may affect multiterritory perforator flap survival according to the effects of autophagy on ischemia/reperfusion injury.

In this study, we analyzed the role of HIF-1α-induced autophagy on multiterritory perforator flap survival in rats, and also elucidated whether HIF-1α participates in the regulation of cell death under ischemia/reperfusion injury. Elucidation of the function of HIF-1α-induced autophagy in the perforator flap during stress conditions may lead to new strategy build up for the improvement of flap survival.

## Results

### Effect of dimethyloxalylglycine (DMOG) and 3-methyladenine (3-MA) on perforator flap survival

**Figure 1:**
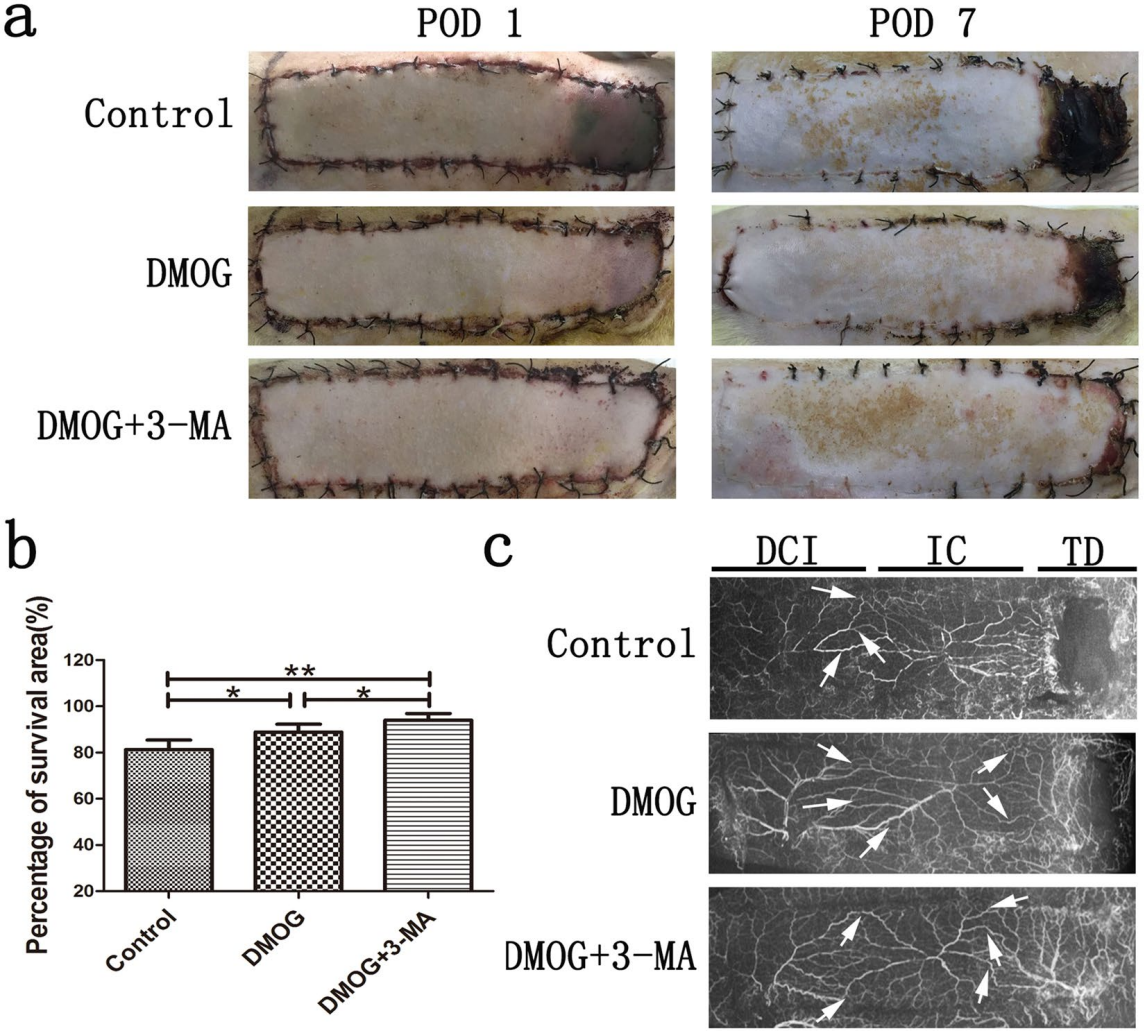
Effect of DMOG and 3-MA on perforator flap survival. (**a**) Postoperative view of DCIA-flap on postoperative days (PODs) 1 and 7 in the control, DMOG and DMOG + 3-MA groups respectively. Necrosis occurred in the thoracodorsal vessel territory. (**b**) Histogram of DCIA-flap survival rate (%) in the control group (81.29 ± 4.20), DMOG group (88.88 ± 3.43) and DMOG + 3-MA group (94.02 ± 2.84). (**c**) Postoperative DCIA-flap angiography showed vascular territory of lateral thoracodorsal (TD), posterior intercostal (IC) and deep circumflex iliac (DCI). White arrows denoted the dilated choke vessel. The vasculature was invisible in the necrosis area. *P < 0.05. **P < 0.001.

In this study, DMOG was used to induce the expression of HIF-1α according to the previous study^8^. After flap surgery, all rats survived without any postoperative infection, and the boundary between survival and necrosis areas was clearly demarcated in each rat on postoperative day (POD) 7 (Fig. 1a). The survival rate (%) of control group was 81.29 ± 4.20 and demonstrated significant difference compared with the other two groups (Fig. 1b; DMOG group: 88.88 ± 3.43, DMOG + 3-MA group: 94.02 ± 2.84, all P < 0.05). The survival difference between DMOG group and DMOG + 3-MA group also showed statistical significance (Fig. 1b; P < 0.05). DMOG’s activity on improving the flap survival was further enhanced by co-administration of autophagy inhibitor, 3-MA.

Among the three groups, the vasculature showed an excellent filling with contrast agent in the DMOG + 3-MA group, which was in accordance with its survival area, while the vasculature in the lateral thoracodorsal (TD) angiosome was unclear in whole or part in the other two groups (Fig. 1c). In the DMOG + 3-MA group, angiography showed obvious dilated choke vessel at choke vessel zone (CVZ), which was located between the TD and posterior intercostal (IC) angiosome.

### Effect of HIF-1α on autophagy expression in perforator flap

**Figure 2:**
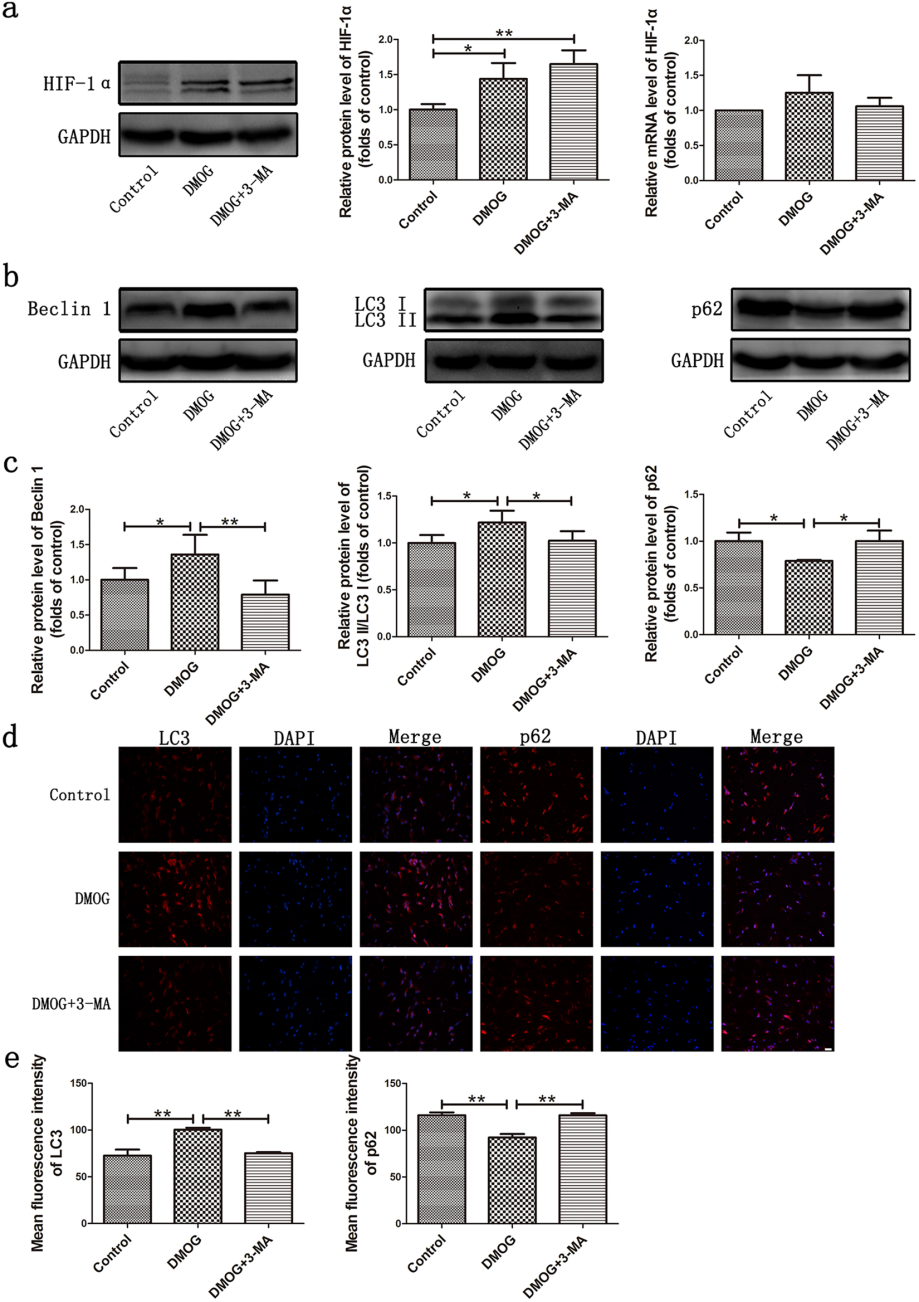
Effect of HIF-1α on autophagy expression in perforator flap. (**a**) The protein and mRNA level of HIF-1α were assessed by western blot analysis and real-time PCR respectively. HIF-1α protein expression was evaluated by optical density analysis, normalized to GAPDH and calculated as the folds of control. Cropped blots are used here and the full-length gel images are available in Supplementary Fig. 1. HIF-1α mRNA level was calculated by Delta-Delta CT method. (**b**) The protein expression of Beclin 1, LC3 and p62 as assessed by Western blot analysis. The band intensity was normalized to GAPDH and calculated as the folds of control. Cropped blots are used here and the full-length gel images are available in Supplementary Fig. 1. (**c**) Densitometry results of Beclin 1, ratio of LC3 II to LC3 I and p62 in the three groups. (**d**) Immunofluorescence staining showed the expression LC3 and p62. Scale bar represents 20μm. (**e**) Analysis of LC3 and p62 fluorescence intensity. LC3: Control group: 72.5 ± 6.6, DMOG group: 100.4 ± 2.1, DMOG + 3-MA group: 75.1 ± 1.2. p62: Control group: 116.0 ± 3.1, DMOG group: 92.2 ± 3.7, DMOG + 3-MA group: 116.0 ± 2.2. *P < 0.05. **P < 0.001.

To investigate the expression level of autophagy after DMOG administration, western blotting analysis or immunofluorescence staining of autophagic proteins Beclin 1, LC3 and p62 were performed. Even though the expression of HIF-1α mRNA showed no change after DMOG administration, HIF-1α protein was significantly increased in the DMOG and DMOG + 3-MA groups (Fig. 2a; P < 0.05). Western blot analyses showed that Beclin 1 and the ratio of LC3 II to LC3 I were significantly up-regulated and were accompanied by the significant up-regulation of HIF-1α (Fig. 2b,c; P < 0.05). Immunofluorescence staining demonstrated accumulation of LC3 in the flap and showed significant differences between the control group and DMOG group (Fig. 2d,e; P < 0.001). All these results suggested the activation of autophagy and generation of autophagosomes in the flap postoperatively. p62 was localized to autophagosomes and degraded in the lysosomes, which in turn acts as a marker of autophagic flux. In the DMOG group, both western blot analysis and immunofluorescence staining showed that the expression of p62 was lower than that in the control group (Fig. 2b,c,d,e; P < 0.05). However, in the DMOG + 3-MA group, co-administration of autophagic inhibitor, 3-MA, inhibited HIF-1α-induced autophagy, resulting in the down-regulation of Beclin 1, declined ratio of LC3 II to LC3 I and up-regulated p62 (Fig. 2b,c,d,e; P < 0.05).

### Effect of HIF-1α-induced autophagy on vascularization in perforator flap

**Figure 3:**
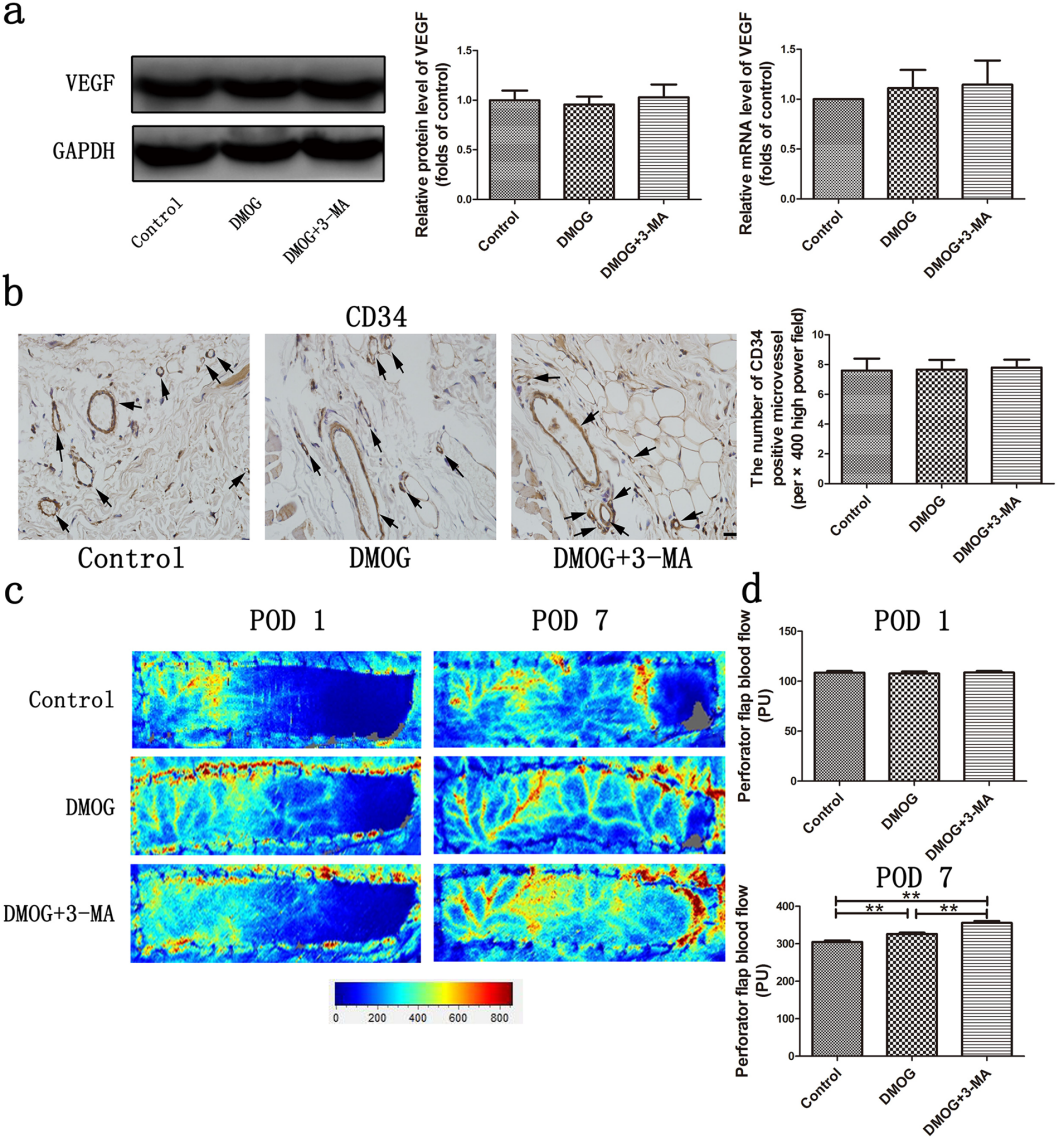
Effect of HIF-1α-induced autophagy on vascularization in perforator flap. (**a**) The protein and mRNA level of VEGF were assessed by western blot analysis and real-time PCR respectively. VEGF protein expression was evaluated by optical density analysis, normalized to GAPDH and calculated as the folds of control. Cropped blots are used here and the full-length gel images are available in Supplementary Fig. 1. The relative VEGF mRNA level was calculated by Delta-Delta CT method. (**b**) Immunohistochemistry staining showed CD34 positive microvessels as marked by black arrows and histogram of mean microvascular density (per × 400 high power field). Control group: 7.60 ± 0.80, DMOG group: 7.67 ± 0.64, DMOG + 3-MA group: 7.80 ± 0.53. Scale bar represents 20μm. (**c**) Full field laser Doppler perfusion images of DCIA-flap on postoperative days (PODs) 1 and 7. Red denoted high perfusion, blue denoted low perfusion and gray denoted no perfusion. Scale bar shows the color for the perfusion value. (**d**) The perfusion value was measured at choke vessel zone between the lateral thoracodorsal and posterior intercostal territory according to the mark made during the flap surgery. On POD 1: Control group: 108.6 ± 1.6, DMOG group: 107.7 ± 2.1, DMOG + 3-MA group: 108.7 ± 1.6. On POD 7: Control group: 305.0 ± 3.7, DMOG group: 326.2 ± 3.5, DMOG + 3-MA group: 356.0 ± 4.8. **P < 0.001.

On POD 1, the differences of both VEGF mRNA and protein expression were not significant among the three groups (Fig. 3a; all P > 0.05), and immunohistochemistry staining also showed no significant differences in the mean microvascular density (MVD), (per × 400 high power field) among the three groups (Fig. 3b; control: 7.60 ± 0.80, DMOG: 7.67 ± 0.64, DMOG + 3-MA: 7.80 ± 0.53, all P > 0.05). In accordance with these results, the laser Doppler perfusion images showed that the flap perfusion (perfusion units, PU) differences were not significant at CVZ on POD 1 (Fig. 3c,d; control: 108.6 ± 1.6, DMOG: 107.7 ± 2.1, DMOG + 3-MA: 108.7 ± 1.6, all P > 0.05). All these results demonstrated that angiogenesis was not affected by HIF-1α up-regulation or inhibition of HIF-1α-induced autophagy in the deep circumflex iliac (DCI) artery perforator flap (DCIA-flap) on POD 1. Even though DMOG could improve blood supply at CVZ on POD 7 compared with that of control group, DMOG + 3-MA could further enhance flap perfusion (Fig. 3c,d; control: 305.0 ± 3.7, DMOG: 326.2 ± 3.5, DMOG + 3-MA: 356.0 ± 4.8, all P < 0.001).

### Effect of HIF-1α-induced autophagy on apoptosis in perforator flap

**Figure 4:**
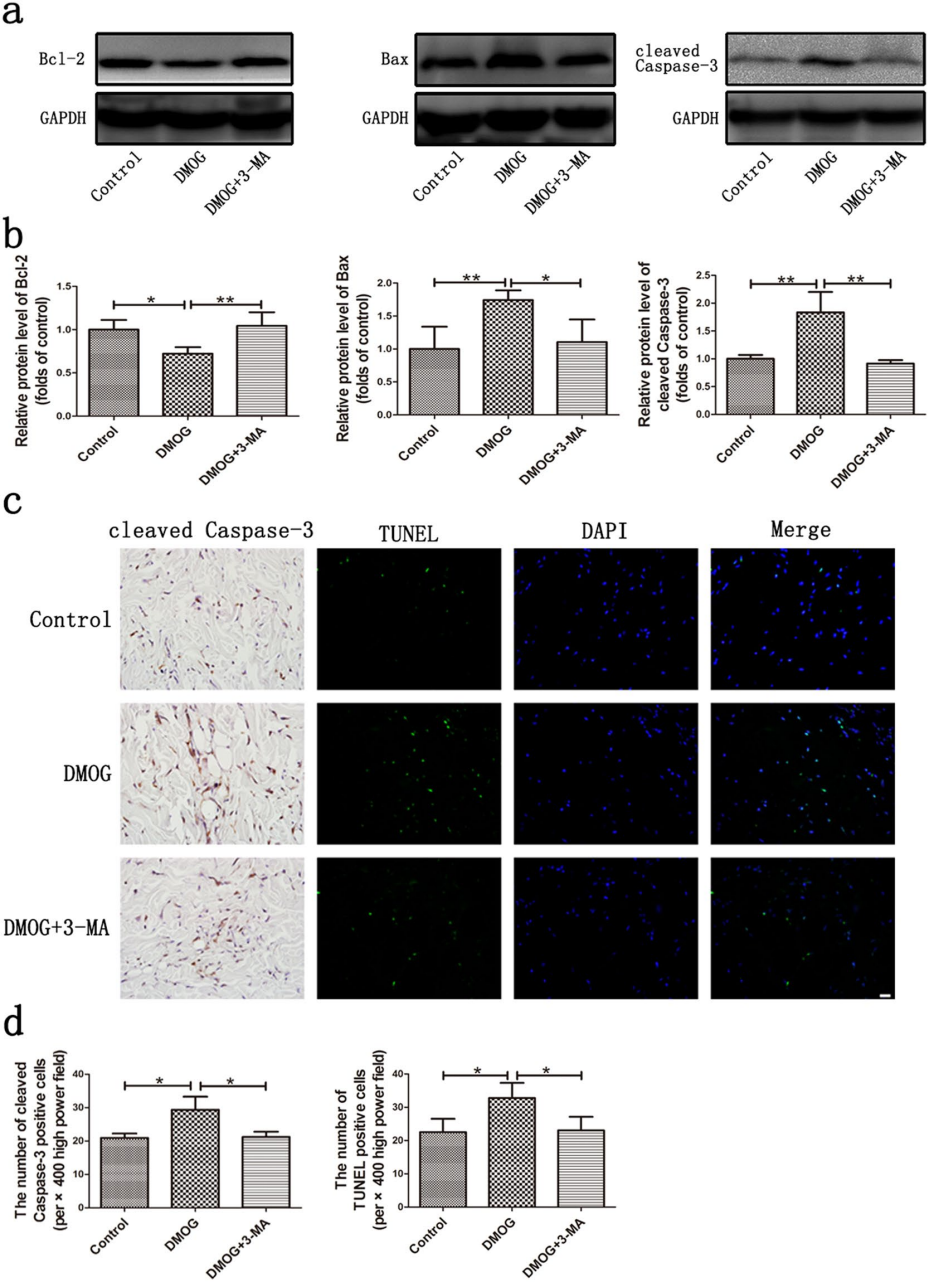
Effect of HIF-1α-induced autophagy on apoptosis in perforator flap. (**a**) The protein expression of Bcl-2, Bax and cleaved Caspase-3 were assessed by western blot analysis. Cropped blots are used here and the full-length gel images are available in Supplementary Fig. 1. (**b**) Histogram of relative protein levels of Bcl-2, Bax and cleaved Caspase-3. Bcl-2, Bax and cleaved Caspase-3 band intensity were normalized to their GAPDH and calculated as the folds of control. (**c**) Immunohistochemistry staining of cleaved Caspase-3 and TUNEL staining. Scale bar represents 20μm. (**d**) The number of cleaved Caspase-3 and TUNEL positive cells (per × 400 high power field). Cleaved Caspase-3: Control group: 20.93 ± 1.29, DMOG group: 29.33 ± 3.95, DMOG + 3-MA group: 21.20 ± 1.59. TUNEL: Control group: 22.53 ± 3.97, DMOG group: 32.8 ± 4.50, DMOG + 3-MA group: 23.07 ± 4.06. *P < 0.05. **P < 0.001.

In this study, we investigated the apoptotic protein expression, including Bcl-2, Bax and cleaved Caspase-3, and TUNEL staining to determine the relationship between HIF-1α-induced autophagy and apoptosis in DCIA-flap postoperatively. Bcl-2 is an anti-apoptotic protein, while Bax is an apoptotic protein and cleaved Caspase-3 participates in the process of apoptosis^14,15^. HIF-1α-induced excessive autophagy in the DMOG group, and was accompanied by the activation of apoptosis. The levels of Bax and cleaved caspase-3 were increased, while Bcl-2 was decreased on POD 1 in DMOG group compared with that of control group (Fig. 4a,b; all P < 0.05). On the other hand, the increased Bax and cleaved caspase-3 levels and decreased Bcl-2 levels were reversed by 3-MA in the DMOG + 3-MA group (Fig. 4a,b; all P < 0.05). Besides, the number of cleaved Caspase-3 positive cells and TUNEL positive cells were increased in the DMOG group (Fig. 4c,d; all P < 0.05), while the differences between the control and DMOG + 3-MA groups were not significant (Fig. 4c,d; all P > 0.05). All these results suggested that HIF-1α-induced autophagy resulted in the activation of apoptosis. Inhibition of autophagy could down-regulate the expression of apoptosis.

### Effect of HIF-1α-induced autophagy on oxidative stress in perforator flap

**Figure 5:**
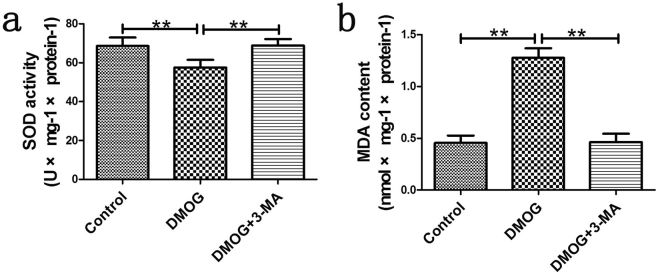
Effect of HIF-1α-induced autophagy on oxidative stress in perforator flap. (**a**) Superoxide dismutase (SOD) activity (U × mg^−1^ × protein^−1^). Control group: 68.64 ± 4.34, DMOG group: 57.60 ± 3.92, DMOG + 3-MA group: 68.81 ± 3.38. (**b**) Malondialdehyde (MDA) content (nmol × mg^−1^ × protein^−1^). Control group: 0.45 ± 0.07, DMOG group: 1.28 ± 0.09, DMOG + 3-MA group: 0.46 ± 0.08. **P < 0.001.

On POD 1, we measured superoxide dismutase (SOD) activity and malondialdehyde (MDA) content to evaluate oxidative stress. Increased reactive oxygen species (ROS) could consume SOD and augment the production of MDA. The SOD (U × mg^−1^ × protein^−1^) was 68.64 ± 4.34 in the control group, 57.60 ± 3.92 in DMOG group and 68.81 ± 3.38 in the DMOG + 3-MA group. Only SOD in the DMOG group was much lower than the other two groups (Fig. 5a; all P < 0.001). The mean level of MDA (nmol × mg^−1^ × protein^−1^) was 0.45 ± 0.07, 1.28 ± 0.09 and 0.46 ± 0.08 in the control, DMOG and DMOG + 3-MA groups, respectively. The MDA content demonstrated significant differences between the DMOG and control groups (Fig. 5b; P < 0.001), and the DMOG and DMOG + 3-MA groups (Fig. 5b; P < 0.001). These results suggested increased oxidative stress accompanied by the up-regulated expression of autophagy in the DMOG group, while autophagy inhibitor could reverse this trend in the DMOG + 3-MA group.

## Discussion

With increasing knowledge on skin blood supply and survival mechanism of the perforator flap^2,20^, more and more surgeons adopted perforator flap in reconstructive and plastic surgeries. After surgery, the resistant choke vessels that connected the anatomic and dynamic territory demonstrated a reduced caliber, which remodeled and developed into true anastomosis without resistance^21,22^. The remodeling process is termed as arteriogenesis, which is referred to as preexisting channel remodeling or de novo formation of arteries, and is responsible for the adjacent vascular territory survival^21,23^. There was a complete vasculature network in the DCIA-flap, whereas the choke vessel between the dynamic and potential territory could not dilate postoperatively. The pre-existing vasculature of potential territory could not deliver oxygen and nutrients to the potential territory, which resulted in necrosis^3,21^. Thus, some authors used delayed procedures and vascular supercharge procedures to improve the flap survival^24,25^. Even though angiogenesis and arteriogenesis were distinctly different mechanisms of neovascularization, angiogenesis was also an effective way to improve flap survival^8,26^. Considering the ischemic situation, neovascularization improvement distinctly played a positive effect on flap survival.

Compared with skin graft suffering from hypoxia only, postoperative flap that suffers from both ischemia and reperfusion injury demonstrated the production of ROS, inflammation, apoptosis and so on^27,28^. Till date several authors have reported effective therapeutic strategies for the inhibition of ischemia/reperfusion injury^27–29^. Among them, usage of hyperbaric oxygen on inhibition of ischemia/reperfusion injury was accepted both clinically as well as in experimental studies^30,31^. The flap survival was improved further by combining the treatment of vascular growth factor and hyperbaric oxygen^32,33^. As mentioned above, the final result of flap was determined by the combined pathophysiologic action of ischemia and reperfusion injury.

DMOG is a HIF prolyl hydroxylases inhibitor and induces the expression of HIF-1α^8,34^. Consistent with the previous studies^8–10^, our study demonstrated the up-regulation of HIF-1α expression improved skin perfusion and promoted DCIA-flap survival on POD 7. However, the expression of VEGF, MVD and skin perfusion were not affected by HIF-1α on POD 1. Angiogenesis is caused due to complicated and comprehensive result of various factors. Examples include the involvement of nitric oxide synthase in governing the vascular tone, proliferation of cells, such as endothelial cells and endothelial progenitor cells, growth factors, growth inhibition factors and their receptors, and matrix metabolism, including plasminogen activator receptors and inhibitors, matrix metalloproteinases and collagen prolyl hydroxylase^5,35,36^. Even though HIF-1α plays a key role in the process of angiogenesis, it is a gradual process and needs time to regulate protein-signaling cascade^5,37^. During the process of angiogenesis, the induced VEGF activates a negative-feedback mechanism of Dll4/Notch pathway, which in turn inhibits vascular branching, and promotes maturation of sprouted microvessel. The Dll4/Notch signal is activated by an up-regulated VEGF expression, but the induced Dll4/Notch pathway inhibits the expression of VEGF in turn^38–40^. This might explain as to why the VEGF expression, MVD and flap perfusion were not significantly different among the three groups on POD 1 in our study. Besides these, few experts reported that HIF-1α could induce endothelial progenitor cells proliferation and recruitment in the ischemic area, and the recruited endothelial progenitor cells was very important in the process of neovascularization, especially for the aged or diabetic mice^9,10,41^.

HIF-1α’s participation in the process of vascularization is not a new topic, but few authors recently proposed the induction of autophagy by HIF-1α^11–13^. Autophagy has a dual role, either beneficial or detrimental in the cell survival, which depends on both the burden of intracellular substance as well as the cellular autophagic capacity^14^. Nishida *et al*. reported that either insufficient or excessive autophagy was detrimental^15^. During the process of autophagy, degradation of damaged or dysfunctional proteins, lipids and organelles generates fatty acid and amino acids for recycling, which consequently promoted cell survival during stress and damage. While under excessive expression of autophagy, some functional components are consumed as well. Thus, the excessive autophagy plays a detrimental effect and promotes cell death^14,17^. For example, some experts reported that an excessive increase in autophagy could enhance tissue injury, apoptosis and production of ROS^17,42,43^.

Apoptosis is a form of programmed cell death and several studies have demonstrated clear mechanism. The relationship between apoptosis and autophagy was investigated in different studies. Basal autophagy can suppress apoptosis and maintain homeostasis, while both insufficient and excessive autophagy can induce apoptosis^15,44^. In this study, we used SOD activity and MDA content to evaluate oxidative stress. SOD is an enzymatic antioxidant agent for detoxifying ROS, and MDA is a product of lipid peroxidation that is used to estimate ROS. Shouval and Elazar found mitochondria as the production house of ROS and the ROS could induce autophagy, which in turn cleared the damaged mitochondria and proteins^45^. Whereas Vande *et al*. suggested an increased production of ROS during the process of excessive autophagy^43^.

Thus, the effect of autophagy varies in different diseases, even in different periods of one specific disease model. Very few studies have reported the effect of autophagy on flap survival^46^. In our study, we found that HIF-1α could induce the activation of autophagy on POD 1, which was accompanied by the augmentation of apoptosis and oxidative stress. Inhibition of HIF-1α-induced autophagy could inhibit apoptosis and relieve oxidative stress injury. Even though the flap survival was still increased in the DMOG group on POD 7 compared with control group, the flap survival was further enhanced by autophagy inhibitor. In our study, HIF-1α showed a positive effect on the perforator flap survival, but HIF-1α also promoted apoptotic expression and oxidative stress injury. As described above, ischemia and reperfusion injury together determined the result of flap postoperatively. We suggested the beneficial effect of HIF-1α’s participation during vascularization was superior to the detrimental effect of HIF-1α-induced autophagy. Therefore, the results presented above confirmed that HIF-1α could improve flap survival, and the survival rate was further enhanced by co-administration of autophagy inhibitor.

In conclusion, HIF-1α could induce autophagy in the multiterritory perforator flap, while the increased autophagy activated apoptosis, augmented oxidative stress and played a detrimental effect on perforator flap survival at the early postoperative stage. No matter whether HIF-1α-induced autophagy was inhibited or not, it showed no effect on the perforator flap vascularization on POD 1. But inhibition of HIF-1α-induced autophagy could suppress apoptosis, relieve oxidative stress and further enhanced perforator flap survival. The extent of ischemia and reperfusion injury together determined the fate of perforator flap.

## Materials and Methods

This study was approved by the Animal Research Committee of Wenzhou Medical University (wydw 2014–0015). Ninety nine male Sprague Dawley rats weighted 250 g to 300 g were purchased from Experimental Animal Center of Wenzhou Medical University (License no. SCXK [ZJ] 2015–0001). All rats received humane care in compliance with the National Institutes of Health Guidelines for the Care and Use of Laboratory Animals. Rats were housed in separate cages with free access to food and water at appropriate temperature (25 °C).

### Flap model and surgical procedures

Rats were anesthetized using 3% sodium pentobarbital (60 mg/kg) via intraperitoneal injection and an additional dose was given during the procedure if necessary. Before surgery, dorsal fur was removed with an electric shaver and depilatory cream. A DCIA-flap was performed on the right side of each rat as reported previously^26^. In this flap, there were three vascular territories, the DCI, IC and TD. In this flap model, the DCI angiosome is an anatomic territory, IC angiosome is a dynamic territory and TD angiosome is a potential territory. The flap position was standardized using bony land marks on the rat dorsum, and was about 2.5 × 11 cm. The flap was elevated beneath the pannculus carnosus. After elevation, the CVZ between TD and IC was identified by transillumination. Then the TD and IC were ligated, and the flap was sutured back into its original site.

### Drug administration

The rats were randomly divided into three groups with 33 in each group. The DMOG + 3-MA group received intraperitoneal injection of DMOG at a dosage of 40 mg/kg (dissolved in 1 ml saline) body weight at 48 hours and 1 hour before procedure and at 48 hours after the procedure, and 3-MA at a dosage of 10 mg/kg (dissolved in 1 ml saline) 1 hour before DMOG every time^8,34,42,47^. The DMOG group received DMOG and equal volume of saline, and the control group received comparative volume of saline at the same time as mentioned above.

### Flap viability

On POD 7, high photographs of the DCIA-flap were obtained using a digital camera to evaluate the flap viability (n = 6). Image-Pro Plus imaging software (ver. 6.0; Media Cybemetics) was used to determine the survival area, which was measured as the percentage of the total flap area.

### Flap angiography

On POD 7, rats underwent whole-body angiography after the survival evaluation (n = 6). Lead oxide-gelatin (50 ml/kg) was perfused into the common carotid artery via a 22-gauge silicone rubber catheter. After 24 hours of fixation, the flap was harvested and radiographed using a soft X-ray machine (54kVp, 40 mA, 100 s exposure).

### Western blot analysis

On POD 1, skin samples (n = 6) from CVZ were collected and stored at −80 °C before Western blot analysis. Total protein was extracted using RIPA lysis buffer, supplemented with protease inhibitor (Beyotime Biotechnology, China). After homogenization and centrifugation of the samples, BCA Protein Assay Kit (Beyotime Biotechnology, China) was used to determine the protein concentrations of the supernatant. Proteins were fractionated by sodium dodecyl sulfate-polyacrylamide gels and transferred onto the PVDF membranes. After blocking with 5% non-fat milk or bovine serum albumin for 2 hours at room temperature, the membranes were incubated overnight with primary antibodies HIF-1α (1:200, Abcam), VEGF (1:1000, Abcam), Beclin 1 (1:1000, Cell Signaling Technology), p62(1:1000, Abcam), LC3 (1:1000, Sigma), Bcl-2 (1:1000, Cell Signaling Technology), Bax (1:1000, Cell Signaling Technology), cleaved Caspase-3 (1:1000, Cell Signaling Technology) and GAPDH (1:2000, Bioworld Technology) at 4 °C. Then the membranes were incubated with goat-anti-rabbit or goat-anti-mouse secondary antibodies for 2 hours, followed by detection with ECL plus reagent kit (Thermo Fisher Scientific, Rockford, IL). Finally, the band intensity was quantified using the Image Lab software (ver.5.2, Bio-Rad).

### Real-time quantitative PCR analysis

Total RNA was isolated from skin flap tissues (n = 3) within the CVZ using Trizol (Invitrogen) and RNeasy kit (Qiagen), and cDNAs were synthesized using Superscript III (Invitrogen) and oligo-dT (Invitrogen). The primer sequences are shown in Table 1. Amplification was performed as follows: 95 °C for 2 minutes, 40 cycles at 95 °C for 10 seconds, 60 °C for 30 seconds, and 70 °C for 45 seconds, and finally 65 °C for 5 min. Relative expression of mRNA was standardized against β-actin by Delta-Delta CT method^48^. Each specimen was measured three times and the mean value was used.

**Table 1:** Primers sequence for real-time PCR.

Gene	Primer sequence
HIF-1α Forward	AGCAATTCTCCAAGCCCTCC
HIF-1α Reverse	GCTGTCCGACTGTGAGTACC
VEGF Forward	CAAACCTCACCAAAGCCAGC
VEGF Reverse	ACGCGAGTCTGTGTTTTTGC
β-actin Forward	AGGGAAATCGTGCGTGAC
β-actin Reverse	CGCTCATTGCCGATAGTG

### Immunofluorescence staining

Skin specimens (n = 3) from CVZ were fixed in 4% paraformaldehyde for 24 hours, dehydrated in sucrose solution series, embedded in OCT and sectioned into 5μm slices on POD 1. After permeabilizing with 0.3% Triton X-100 and blocking with 10% goat serum in PBS for 1 hour at room temperature, these slides were incubated with primary antibody p62 (1:150, Abcam) and LC3 (1:100, Sigma) at 4 °C for overnight. Under darkness, the sections were incubated with TRITC conjugated goat anti-rabbit IgG (1:100, Bioworld Technology) for 1 hour, followed by incubation with DAPI for 2 minutes at room temperature. After that, the sections were sealed with a coverslip and evaluated under a fluorescence microscope (Olympus Corp) at × 400 magnification. The fluorescence intensity of ten positive cells was evaluated under five high expression fields in each section and the mean fluorescence intensity was used to present the protein expression level.

### Immunohistochemistry staining

On POD 1, samples (n = 3) from CVZ were fixed in 4% paraformaldehyde for 24 hours, dehydrated in alcohol, embedded in paraffin and sectioned into 5μm slices. The sections were deparaffinized through xylene and rehydrated via a grade set of ethanol. Antigen retrieval was then carried out for 10 minutes at 100 °C. After that the sections were immersed in 3% H_2_O_2_ to inhibit endogenous peroxidase activities and incubated in 10% normal goat serum to saturate non-specific sites. After overnight incubation at 4 °C with CD34 (1:50, Abcam) and cleaved Caspase-3 (1:200, Abcam), horseradish peroxidase labeled goat anti-rabbit antibody was used as a secondary antibody, and diaminobenzidine as chromogen. Finally, the sections were counterstained with hematoxylin. The sections were imaged at × 400 magnification using a DP2-BSW image-acquisition system (Olympus Corp). We counted the number of CD34 positive microvessels and cleaved Caspase-3 positive cells in five dense fields in each specimen.

### Laser Doppler perfusion image

Full field laser Doppler perfusion images (n = 6) were obtained using a laser Doppler instrument (Moor Instruments, Axminster, UK) in a warm and quiet environment under anesthesia. The perfusion images were processed to provide a color-coded living flux image and the blood flow at CVZ was measured on PODs 1 and 7. The measurement in each rat was repeated three times and the mean value was used.

### TUNEL staining

TUNEL staining was performed following the DeadEnd^TM^ Fluorometric TUNEL System (Promega) protocol. Briefly, the paraffin-embedded section (n = 3) was deparaffinized by xylene, rehydrated through graded ethanol and permeabilized via 0.3% Triton X-100. Then the fluorescein-12-dUTP was used to label DNA stand breaks and DAPI to label the nucleus. The sections were imaged at × 400 magnification using a DP2-BSW image-acquisition system (Olympus Corp). We counted the number of TUNEL positive cells in five dense fields and the mean value was used.

### SOD activity and MDA content

SOD activity and MDA content were used to assess oxidative stress in the flap according to the kit protocol from Nanjing Jiancheng Biology Institution (Nanjing, China). On POD 1, specimens (n = 6) from CVZ were weighed, homogenized and centrifuged at 5% dilution with saline. SOD activity was identified following xanthine oxidase method and MDA content was confirmed through reaction with thiobarbituric acid at 95 °C according to the previous report^26^.

### Statistical analyses

Statistical analyses were performed by one-way ANOVA using SPSS software version 19.0 (SPSS, Chicago, IL). All data were presented as mean ± standard deviation (SD). A two-tailed p-value < 0.05 was considered to be statistically significant.

## Electronic supplementary material

Supplementary Fig. 1
