# Supplementary material for: Detrimental effect of Hypoxia-inducible factor-1α-induced autophagy on multiterritory perforator flap survival in rats
Source: Sci Rep. 2017 Sep 18;7:11791. doi: 10.1038/s41598-017-12034-x (PMC5603514; doi:10.1038/s41598-017-12034-x)
Supplement: Supplementary file 1 — Supplementary Fig. 1 [file 41598_2017_12034_MOESM1_ESM.doc]

Detrimental effect of Hypoxia-inducible factor-1α-induced autophagy on multiterritory perforator flap survival in rats.

Long Wang^1^ e-mail: wanglong0927@yeah.net

Zhicheng Jin^1^ e-mail: zhichengjinsaber@126.com

Jieke Wang^1^ e-mail: 756390005@qq.com

Shao Chen^1^ e-mail: 1195134432@qq.com

Li Dai^1^ e-mail: 1131812152@qq.com

Dingsheng Lin^1^ e-mail: lindingsheng@gmail.com

Lingfeng Wu^2^  e-mail: wlf0626@126.com

Weiyang Gao^1*^ e-mail: weiyanggaoi@126.com

**Affiliations**1Department of Hand and Plastic Surgery, The Second Affiliated Hospital and Yuying Children’s Hospital of Wenzhou Medical University, Wenzhou, China

2Department of Orthopedics, The Fifth Affiliated Hospital and Central Hospital of Lishui City of Wenzhou Medical University, Lishui, China,

***Corresponding author**Weiyang Gao, MD

Department of Hand and Plastic Surgery, The Second Affiliated Hospital and Yuying Children’s Hospital of Wenzhou Medical University, Wenzhou, China

109 West Xue Yuan Road, Lu Cheng District, Wenzhou 325000, China
Tel: +86 577 88002812
Fax: +86 577 88002812
Email: weiyanggaoi@126.com


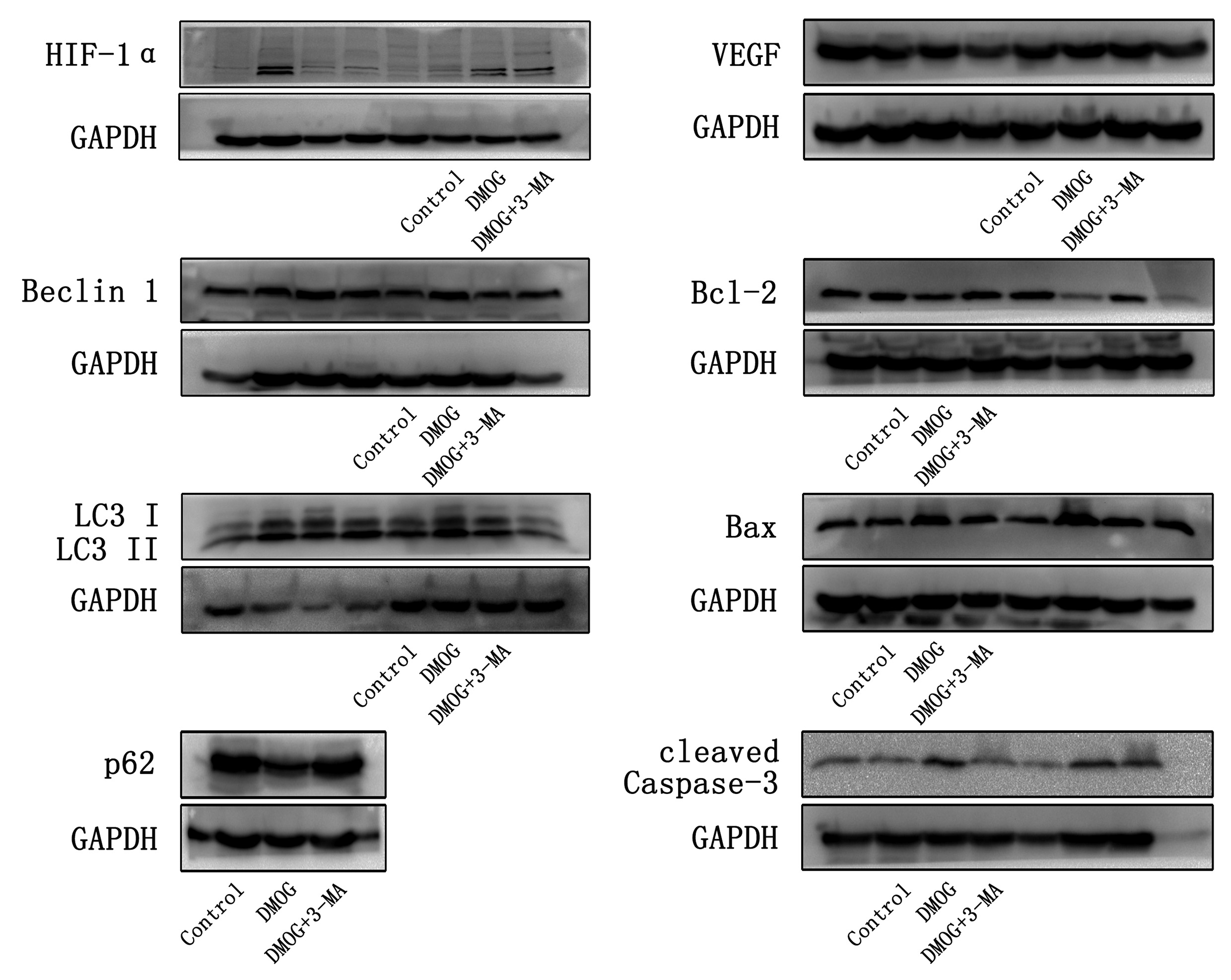


Supplementary Fig. 1 The full-length gel images of HIF-1α, Beclin 1, LC3, p62, VEGF, Bcl-2, Bax, cleaved Caspase-3 and GAPDH.
